# Redox Dependent Modifications of Ryanodine Receptor: Basic Mechanisms and Implications in Heart Diseases

**DOI:** 10.3389/fphys.2018.01775

**Published:** 2018-12-06

**Authors:** Roman Nikolaienko, Elisa Bovo, Aleksey V. Zima

**Affiliations:** Department of Cell and Molecular Physiology, Loyola University Chicago, Maywood, IL, United States

**Keywords:** heart, Ca signaling, ryanodine receptor, sarcoplasmic reticulum, oxidative stress

## Abstract

Heart contraction vitally depends on tightly controlled intracellular Ca regulation. Because contraction is mainly driven by Ca released from the sarcoplasmic reticulum (SR), this organelle plays a particularly important role in Ca regulation. The type two ryanodine receptor (RyR2) is the major SR Ca release channel in ventricular myocytes. Several cardiac pathologies, including myocardial infarction and heart failure, are associated with increased RyR2 activity and diastolic SR Ca leak. It has been suggested that the increased RyR2 activity plays an important role in arrhythmias and contractile dysfunction. Several studies have linked increased SR Ca leak during myocardial infarction and heart failure to the activation of RyR2 in response to oxidative stress. This activation might include direct oxidation of RyR2 as well as indirect activation via phosphorylation or altered interactions with regulatory proteins. Out of ninety cysteine residues per RyR2 subunit, twenty one were reported to be in reduced state that could be potential targets for redox modifications that include S-nitrosylation, S-glutathionylation, and disulfide cross-linking. Despite its clinical significance, molecular mechanisms of RyR dysfunction during oxidative stress are not fully understood. Herein we review the most recent insights into redox-dependent modulation of RyR2 during oxidative stress and heart diseases.

## Excitation-Contraction Coupling

**FIGURE 1:**
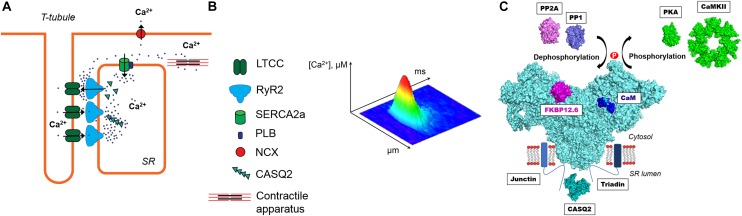
**(A)** The main components of intracellular Ca regulation in ventricular myocytes. A Ca release unit is formed by a cluster of ryanodine receptors (RyR2) in the junctional sarcoplasmic reticulum (SR) and L-type Ca channels (LTCC) in the sarcolemma. During systole, inward Ca current through the LTCC activates RyR2 by the mechanism called Ca-induced Ca release (CICR). A global Ca transient causes activation of the contractile apparatus and thus myocyte contraction. During diastole, cytosolic Ca is pumped back into the SR by the Ca-ATPase (SERCA) and extruded from the cell by the Na-Ca exchanger (NCX). **(B)** A confocal image of diastolic Ca spark. **(C)** The multimolecular RyR2 complex. On the cytosolic side, RyR2 interacts with calmodulin (CaM) and FK-506 binding protein 12.6 (FKBP12.6). The activity of RyR2 is also regulated by two major protein kinases (PKA and CaMKII) and two phosphatases (PP1 and PP2A). At the luminal side, RyR2 is associated with the Ca-sensing complex formed by triadin, junctin and calsequestrin (CASQ2). The molecular representation of the illustrated proteins was created from Protein Data Bank entries (www.rcsb.com, PDB_IDs: 5L1D, 1CPK, 2W2C, 2BCX, 1UP5, 4MOV, 2IAE, 2VAF, 4IQ2).

Regular heart contraction critically depends on precisely-controlled cytosolic Ca regulation during each cardiac cycle. The sarcoplasmic reticulum (SR) Ca release plays a particularly important role in activation of myocyte contraction (Zima et al., 2014). During systole, Ca release from the SR is a result of activation of specialized Ca channels – ryanodine receptors (RyR). These channels are activated by an inward Ca current via L-type Ca channels (LTCCs) during an action potential (AP) (Figure 1A). The mechanism of RyR activation by cytosolic Ca is known as Ca-induced Ca release (CICR) (Fabiato, 1983). In ventricular myocytes, CICR occurs at specialized microdomains where a T-tubule of the sarcolemma closely approaches a junction of the SR forming the dyad. The junctional SR membrane contains clusters of RyRs (Franzini-Armstrong et al., 1999; Hayashi et al., 2009). The activation of a single RyR cluster generates a local increase in cytosolic Ca ([Ca]_i_) called Ca spark (Cheng et al., 1993; Figure 1B). The spatio-temporal summation of thousands of Ca sparks produces the global Ca transient that initiates contraction. During diastole, cytosolic Ca is pumped back into the SR by the Ca-ATPase (SERCA) and extruded from the cell by the Na-Ca exchanger (NCX) (Bers, 2002). The rate at which SERCA and NCX remove Ca from the cytosol determines how quickly cardiac muscle relaxes to allow the heart to fill with blood.

## Ryanodine Receptor Complex

Three isoforms of RyR have been identified. The RyR1 isoform is dominant in skeletal muscles, whereas the RyR2 represents the cardiac RyRs isoform. The RyR3 isoform is found only at low expression levels in certain skeletal muscle types and brain (Lanner et al., 2010; Meissner, 2017). RyR forms a homo tetrameric assembly comprised of four 560 kDa subunits yielding a total molecular weight of 2,300 kDa (Van Petegem, 2015). The RyR2 is not only an ion channel but also a giant scaffolding protein on which several regulatory proteins and enzymes can be assembled (Fill and Copello, 2002; Bers, 2004; Figure 1C). RyR2 forms a multimolecular complex with FK506 binding protein 12.6 (FKBP12.6) and calmodulin (CaM) at the cytosolic side. FKBP12.6 binds to each subunit of RyR2 with high affinity and stabilizes the channel in closed conformation (Brillantes et al., 1994; Timerman et al., 1994; Wehrens et al., 2004). Another small regulatory protein CaM binds RyR with nanomolar affinity, at 1:4 ratio in the absence and presence of Ca (apoCaM and Ca-CaM) (Meissner, 2017). The effect of CaM on RyR activity is isoform specific. At free [Ca] > 1 μM CaM inhibits all three isoforms of RyR, whereas at submicromolar free [Ca] CaM activates RyR1 and RyR3, and inhibits RyR2 (Balshaw et al., 2001; Meissner, 2004). At the luminal side, RyR2 is associated with the complex consisting of triadin-1, junctin and calsequestrin that act together as a luminal Ca sensor (Györke et al., 2004). Other reports suggest RyR2 interactions with junctophilin, homer-1, sorcin and S100A1 (Song D.W. et al., 2011). Additionally, the RyR2 complex comprises enzymes that regulate the channel activity through the interplay of phosphorylation and dephosphorylation by protein kinases and protein phosphatases. Protein kinase A (PKA) phosphorylates RyR2 at serines 2030 and 2808, whereas Ca-CaM dependent protein kinase II (CaMKII) phosphorylates the channel at serine 2814 (Marx et al., 2000; Wehrens, 2004; Xiao et al., 2006). Phosphorylation can be reversed by associated with the RyR2 complex protein phosphatases 1 and 2A (PP1 and PP2A) (Marx et al., 2000).

## RYR2-Mediated SR Ca Leak

The majority of SR Ca release occurs during systole via the mechanism of CICR. During diastole, however, spontaneous openings of RyR2s can produce SR Ca leak (Shannon et al., 2003; Zima et al., 2014). At normal physiological conditions, a large fraction of SR Ca leak occurs as uncoordinated openings of individual RyR2s or spark-independent Ca leak. This leak component can serve as an important protective mechanism against SR Ca overload. (Zima et al., 2010). However, in pathological conditions associated with increased RyR2 activity, the majority of SR Ca leak occur in a form of Ca sparks and Ca waves. By activating the electrogenic NCX, spontaneous Ca waves can generate delayed afterdepolarizations (DADs), an effective trigger of cardiac arrhythmias (Pogwizd and Bers, 2004). Increased diastolic SR Ca leak through RyR2s channel has been also implicated in the development of several cardiac pathologies, including heart failure (HF) (Shannon et al., 2003; Kubalova et al., 2005; Belevych et al., 2007; Bers, 2014). It has been suggested that SR Ca leak contributes to the depressed Ca transients and the reduced SR Ca load in HF. The increased RyR2-mediated Ca leak has been also implicated in the progression of arrhythmogenesis in failing hearts (Blayney and Lai, 2009). It has been suggested that oxidative post-translational modifications in RyR2 may play an important role in the abnormal channel activity and the increased SR Ca leak in many cardiac pathologies (Mochizuki et al., 2007; Terentyev et al., 2008; Belevych et al., 2009, 2011, 2012; Bovo et al., 2018a,b). The functional effect of these redox modifications, including disulfide oxidation, mixed disulfide formation (S-glutathionylation) and S-nitrosylation, will be further discussed in this review.

## ROS Production in Cardiac Muscle

Cardiac muscle contraction strongly depends on ATP synthesis by the mitochondrial electron transport chain (ETC). In addition to energy production, the ETC activity can lead to generation of reactive oxygen species (ROS). During the reduction of molecular oxygen to water some redox centers, in particular complex I and III, in the ETC may leak electrons to oxygen producing superoxide anion (O_2_^-^●) (Turrens, 2003). O_2_^-^● is further converted into hydrogen peroxide (H_2_O_2_●) by superoxide dismutase (SOD) enzymes. Transitional metals can react with H_2_O_2_ with a formation of even stronger oxidant – hydroxyl radical (OH●) (Madamanchi and Runge, 2013). ROS can be also generated by cytochrome P450-based enzymes, xanthine (XO) and NADPH oxidases (NOX) (Zima and Blatter, 2006; Santos et al., 2016). Additionally, cardiomyocytes express two isoforms of nitric oxide (NO●) synthase – endothelial and neuronal NOS (NOS1 and NOS3, respectively) that play an important role in the intracellular signaling (Santos et al., 2011). Besides generation of NO●, NOSs are also responsible for the production of reactive nitrogen species (RNS). Uncoupled NOSs can generate O_2_^-^●, which can be combined with NO● to produce the powerful oxidant peroxinitrite (ONOO^-^●).

Deleterious effects of increased ROS production include lipid peroxidation, protein oxidation, DNA mutagenesis and DNA-protein cross-linking. Therefore, amounts of ROS inside the cell must be tightly controlled by enzymatic and non-enzymatic antioxidant defense mechanisms (Madamanchi and Runge, 2013). These mechanisms comprise catalase, SOD, glutathione peroxidase and vitamins A, C and E (Zima and Blatter, 2006; Santos et al., 2016). O_2_^-^● produced in mitochondria is converted to H_2_O_2_ by SOD, which is further reduced to water by glutathione peroxidase, catalase, peroxiredoxin and thioredoxin systems (Foster et al., 2009). Non-enzymatic defense mechanisms rely on small antioxidant molecules, primarily on the intracellular pool of reduced glutathione (GSH). It has been shown that GSH can directly acts as ROS scavenger. Upon its reaction with ROS, GSH is oxidized into GSSG that can be reduced back to GSH by glutathione reductase (Schafer and Buettner, 2001). Glutathione reductase plays a key role in maintaining the intracellular GSH/GSSG ratio in a proper physiological range (30:1–100:1). GSH also contributes to the maintenance of the cellular redox state by acting as a substrate for glutathione peroxidase (Foster et al., 2009). When the amount of ROS production overwhelms the intracellular anti-oxidant defense, oxygen free-radicals cause damage of DNA, lipids and proteins. These uncontrolled conditions are also known as oxidative stress (Giordano, 2005).

At physiological conditions, however, low levels of ROS and RNS are involved in the cell signaling by inducing discrete, reversible and site-specific protein modification (Heymes et al., 2003; Giordano, 2005; Foster et al., 2009). The redox signaling is based on the ability of ROS/RNS to modulate protein cysteines, leading to S-nitrosylation, S-glutathionylation, and disulfide bond formation. Such redox modifications would affect activity of the proteins involved in different signaling cascades. For example, in cardiac muscle NOX activity and ROS production can be stimulated by growth factors and cytokines, such as angiotensin II, PDGF and TNF-α (Giordano, 2005). In these pathways, ROS act as important second messengers related to inflammatory and stress response. Redox modifications of signaling proteins can be reversed by specific enzymes. Disulfide bridges and mixed disulfides (S-glutathionylation) can be reduced by both thioredoxin and glutaredoxin systems. Additionally, thioredoxin system can reduce S-nitrosothiols. Both glutaredoxin and thioredoxin system utilize the reducing power of NADPH for reduction of their key enzymes – glutathione and thioredoxin reductases (Paulina Donoso and Gina Sánchez, 2013).

In the heart, increased ROS production during oxidative stress has been associated with different cardiac pathologies, including myocardial infarction (MI) and HF. During MI, the re-oxygenation of ischemic region is vital for heart survival. However, re-oxygenation also causes significant myocardium damage which has been linked to the toxic effects of ROS (Zweier and Talukder, 2006). Significant increase in ROS production during ischemia/reperfusion (I/R) arises from the uncoupled mitochondrial ETC as well us upregulation of NOX, NOS and XO resulting in increased generation of $O_{2}^{•-}$ and decreased GSH/GSSH ratio (Zima and Mazurek, 2016). Oxidative stress during cardiac pathologies is commonly associated with increased SR Ca leak through the hyperactive RyR2. Moreover, ROS can activate hypertrophic and pro-apoptotic signaling pathways, leading to myocardial remodeling (Sawyer et al., 2002; Santos et al., 2016). HF is frequently viewed as a condition of chronic oxidative stress (Mak and Newton, 2001). The unbalanced metabolism has been suggested to play an important role in development of HF (Mak and Newton, 2001; Ventura-Clapier et al., 2004; Santos et al., 2011). As the disease progresses, oxidative stress worsens due to the increasing energy demand and workload of the failing heart, thus perpetuating a deleterious cycle (Seddon et al., 2007). Although HF is associated with a large number of complex intracellular changes, the focus of this review is directed at understanding the role of oxidative stress in SR Ca regulation and RyR2 function. To date, RyR2 dysfunction has been characterized by increased phosphorylation and oxidation levels. While functionally important phosphorylation sites on RyR2 have been characterized (Marx et al., 2000; Wehrens, 2004; Xiao et al., 2006), the specific mechanisms of oxidative modifications of RyR2 and their contribution to defective SR Ca cycling remain incomplete.

## Redox Regulation of RYR2

Historically, oxidative stress has been linked to an increase of SR Ca release (Trimm et al., 1986). It has been proposed that oxidation of cysteines in RyR2 causes significant changes in the channel gating (Abramson and Salama, 1989). Out of 90 cysteine residues in the single subunit of RyR2, about 21 are in the free thiol state and available for redox-modifications (Xu, 1998; Donoso et al., 2011). Therefore, it is no surprise that RyR2 has been characterized as the highly redox sensitive ion channel. During oxidative stress, sulfhydryl groups of cysteine residues on RyR2 can be oxidized by ROS producing sulfenic, sulfinic and sulfonic acids (Giles and Jacob, 2002). While there is no evidence for the functional significance of sulfinic and sulfonic acids, sulfenic acid can react with sulfhydryl groups, RNS and GSH, yielding disulfide bridges, S-nitrosylation, S-glutathionylation, respectively (Paulina Donoso and Gina Sánchez, 2013). In general, oxidation of cysteine residues has been suggested to cause RyR2 activation (Suzuki and Ford, 1999; Zima and Blatter, 2006). However, multiple studies have shown that effects of oxidative agents on RyR2 largely depend on experimental conditions (Mi et al., 2015). It has been demonstrated that low concentrations of oxidizing agents activate RyR2, whereas prolonged exposure or high concentrations of oxidants lead to irreversible RyR2 inhibition (Dulhunty et al., 2000). Different cysteine residues have been suggested to play a role in activation or inhibition of RyR2 by oxidative stress.

The role of RyR2 redox modifications in cardiac pathologies has been investigated in numerous studies. It has been shown that the non-selective beta-blocker with antioxidant properties carvedilol is more effective in a treatment of HF than other beta-blockers. In the pacing-induced HF model, carvedilol was able to preserve the cardiac function by stabilizing RyR2 structure. It has been suggested that some of the beneficial effects of carvedilol can be attributed to its ability to prevent oxidation of RyR2. In experiments with administration of the NO^-^●/ONOO^-^● donor SIN-1, carvedilol was able to prevent thiol oxidation in RyR2, presumably by acting as a scavenger of ONOO^-^● (Mochizuki et al., 2007). In the canine model of chronic HF, the increased SR Ca leak has been attributed to redox modification of RyR2 by ROS. Interestingly, reducing agents that target S-nitrosylation and S-glutathionylation failed to reverse SR Ca leak in HF. At the same time, the application of DTT (an agent that can reverse disulfide formation, S-nitrosylation and S-glutathionylation) decreased RyR2-mediated Ca leak, suggesting a central role of disulfide bond formation in this process (Terentyev et al., 2008). Studies of the canine post-MI model with ventricular fibrillation (VF) has also shown increased diastolic SR Ca leak and decreased SR Ca content. Similarly to HF studies, cardiomyocytes isolated from the canine post-MI model were characterized by increased levels of ROS production and RyR2 oxidation (Belevych et al., 2009). Another study of the rabbit I/R model has shown a critical role of RyR2 oxidation in the transition from the positive inotropic to arrhythmogenic effect during β-adrenergic receptor stimulation (Bovo et al., 2018a). Taken together, these data suggest that SR Ca leak due to RyR2 oxidation can be a common mechanism of SR Ca mishandling during different cardiac pathologies. Therefore, reactive cysteines on RyR2 may represent an important target in the development of new therapeutic strategies.

## Identification of RYR2 Redox-Modification Sites

Given the functional significance of redox modifications of RyR, several structural studies were performed in order to allocate and characterize sites of these modifications. In the skeletal isoform RyR1, cysteine 3635 located in the CaM-binding site has been shown to undergo functionally significant S-nitrosylation (Sun et al., 2001). Mass spectroscopy study has identified nine cysteine residues (including cysteine 3635) in RyR1 that were S-alkylated by low doses of the maleamide derivative CPM, suggesting their high-sensitivity to redox modifications (Voss et al., 2004). Other studies have proposed that cysteine 3635 can undergo S-glutathionylation and could be also involved in RyR disulfide cross-linking (Aracena-Parks et al., 2006). Twelve RyR1 cysteine residues, including cysteine 3635, have been proposed to be either S-nitrosylated, S-glutathionylated or to form disulfide bridges. Of those twelve cysteines, nine were shown to be endogenously redox-modified. It has been suggested that cysteine 3635 can form a disulfide bond with cysteine residues in the region 1–2401 of RyR1 (possibly with either cysteine 36, 2326, or 2363). However, the functional significance of these redox-modifications remains unclear yet.

While skeletal RyR1 hyper-reactive cysteines have been characterized, little is known about corresponding redox modifications in the cardiac RyR2. A functional role of RyR2 cysteine 3602 (which corresponds to the hyper-reactive cysteine 3635 in RyR1) in Ca overload-induced Ca release has been studied in HEK293 cells expressing recombinant RyR2. It has been shown that the mutation of RyR2 cysteine 3602 to alanine (C3602A) significantly increased SR Ca fractional release by decreasing termination and increasing activation threshold for CICR. Interestingly, the ability of N-ethylmaleamide (NEM) to increase the activation threshold for CICR for the RyR2 was lost for the C3602A mutant, suggesting functional significance of cysteine 3602 alkylation. However, no difference was observed in the effect of cysteine oxidants on WT and C3602A RyR2 (Mi et al., 2015). Despite ∼70% homology between RyR1 and RyR2, these channels appear to exhibit important structural and functional differences, particularly with respect to redox regulation.

## S-Glutathionylation of RYR2

High pKa (>8.0) of a sulfhydryl group means that the majority of free thiols is protonated under physiological pH (7.0–7.4) and cannot be oxidized. As a result, protein redox modifications require an initial conversion of a sulfhydryl group to a thiolate anion. Proton dissociation from a thiol group is highly dependent on the local microenvironment and can be facilitated by closely located basic amino-acids (Grek et al., 2013). During oxidative stress, spontaneous protein S-glutathionylation may occur via multiple pathways that include thiol-disulfide exchange between a protein thiol and GSSG, a reaction between sulfenic acid or thiyl radical (**-**S●) and GSH, as well as S-glutathionylation induced by RNS. In addition to spontaneous reactions, the rate and efficiency of S-glutathionylation can be enhanced by enzymatic activity of glutathione-S-transferases (GSTs) (Zhang et al., 2018). Because of the ability of glutaredoxin and thioredoxin systems to reverse S-glutathionylation, this redox modification can prevent irreversible oxidation of protein thiol groups into sulfonic acids (Townsend, 2007). However, the addition of large and negatively charged glutathione group may also significantly alter structural and functional properties of a protein. Moreover, two adjacent S-glutathionilated thiols can displace GSH groups with a formation of disulfide bond within the protein (Beer et al., 2004).

**FIGURE 2:**
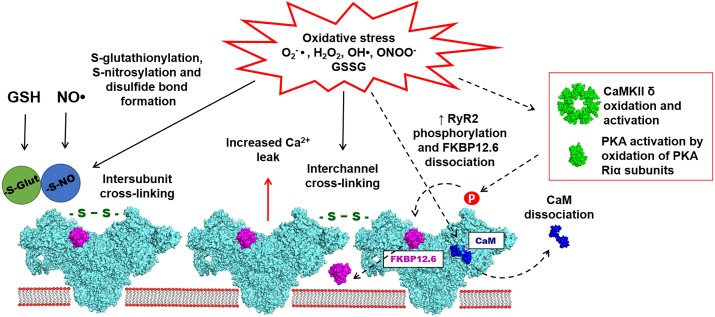
Oxidative stress is associated with the increased accumulation of reactive oxygen and nitrogen species (ROS/RNS), such as O2-●, H_2_O_2_, OH●, and ONOO-●. ROS and RNS have been implicated in redox-dependent post-translational modifications of cardiac RyR2, including S-glutathionylation, S-nitrosylation and disulfide bond formation (marked with solid arrows). Disulfide bond formation also leads to RyR2 cross-linking, which presumably may take place between subunits within one channel or between subunits of different channels. Oxidative stress may also indirectly modulate the activity of RyR2 (marked dashed arrows). Accumulation of ROS/RNS leads to calmodulin (CaM) dissociation from RyR2, potentially caused by the oxidation of RyR2 and/or CaM. Oxidative stress can also activate PKA and CaMKII leading to an increase of RyR2 phosphorylation on Serine 2808 and Serine 2814 sites. It has been also suggested that the PKA-dependent phosphorylation may lead to increased FKBP12.6 dissociation from RyR2. The effects of oxidative stress on RyR2 mostly lead to the increased diastolic SR Ca leak resulting in arrhythmias and contractile dysfunction in variety of cardiac pathologies.

Several studies have provided evidence for the functional role of RyR2 S-glutathionylation during oxidative stress (Figure 2). It has been reported that preconditioning tachycardia increased NOX activity in the SR microsomes isolated from the canine ventricle. The increased NOX activity led to increased RyR S-glutathionylation and elevated systolic SR Ca release (Sánchez et al., 2005). The same effect was described for the SR microsomes isolated from animals preconditioned with exercises. It has been suggested that NOX activation and RyR2 S-glutathionylation during preconditioning may have a protective effect by increasing systolic SR Ca release while limiting diastolic SR Ca leak (Sánchez et al., 2008). In our recent study, we described the effects of oxidized glutathione (GSSG) on SR Ca leak in rabbit ventricular myocytes. We found that an application of GSSG to permeabilized myocytes increased diastolic SR Ca leak and induced RyR2 disulfide cross-linking (Mazurek et al., 2014). HF myocytes were also characterized by elevated GSSG level, RyR2 cross-linking and increased SR Ca leak. Blocking RyR2 cross-linking with the alkylating agent NEM decreased SR Ca leak and prevented depletion of SR Ca load. Based on these results, we suggested that oxidative stress in the failing heart promotes abnormal inter-subunit interactions within the RyR2 complex that increase channel opening leading to the increased SR Ca leak (Bovo et al., 2018a).

Interestingly, some studies suggest an interaction between RyR and GSTs, enzymes that catalyze S-glutathionylation. The members of GST family CLIC-2, GSTO1-1, and GSTM2-2 were identified as endogenous RyR modulators that exert a strong inhibitory effect on RyR2 and weak excitatory effect on RyR1. The isoform-specific effect of GSTM2-2 has been explained by its binding to the RyR2 divergent region 3, not present in RyR1 (Liu et al., 2012) The C-terminal non-catalytic region of the muscle specific GSTM2-2 has been shown to bind to RyR2 independently from N-terminal glutathione transferase activity (Dulhunty et al., 2011). However, it remains unclear whether GSTM2-2 may be involved in RyR2 S-glutathionylation *in vivo*.

## S-Nitrosylation of RYR2

NO● plays an important role in cardiovascular signaling through its involvement in the cGMP-dependent vasodilation pathway. NO● binds to guanylate cyclase (GC) increasing the production of cGMP that activates several cGMP-dependent enzymes, including cAMP phosphodiesterase (PDE). The PDE activation would decrease cAMP level and PKA activity. This, in turn, would cause a decrease in PKA phosphorylation of several proteins, including RyR2, LTCC and phospholamban (PLB). cGMP can also activate cGMP-dependent kinase (PKG). It has been reported that PKG can phosphorylate RyR2 at the CaMKII site (Takasago et al., 1991). However, the functional significance of PKG-mediated RyR2 phosphorylation remains elusive. NO●**-**based signaling reactions can also occur through the NO●**-**dependent redox-modification, known as S-nitrosylation (Lima et al., 2010; Figure 2). The reaction of S-nitrosylation involves a transfer of NO● group to a nucleophilic thiol group yielding S-nitrosothiol (Sun and Murphy, 2010). To initiate S-nitrosylation, thiol groups have to be oxidized (Donoso et al., 2011). It has been shown that intracellular proteins can be effectively nitrosylated by endogenously produced NO● as well by exogenous NO● donors (Hess et al., 2005).

The majority of functional studies of RyR S-nitrosylation have been conducted in lipid bilayer experiments with skeletal and cardiac SR vesicles. It has been shown that NO● donors S-nitrosoglutathione and S-nitrosocysteine can induce S-nitrosylation on each RyR2 subunit at three different sites. S-nitrosylation of RyR2 was associated with an increase of the channel open probability. Interestingly, a comparable RyR2 oxidation of 5-6 thiols per subunit showed no significant effect on the RyR2 function, suggesting a different role of thiol S-nitrosylation and oxidation in the channel activity (Xu, 1998). In another study, S-nitrosothiol reagents increased Ca release from skeletal and cardiac muscle SR vesicles, confirming that RyR S-nitrosylation activates RyR (Stoyanovsky et al., 1997). In both reports, the NO●/ONOO^-^● donor SIN-1 produced a significant RyR activation that could not be reversed by reducing agents, suggesting irreversible thiol oxidation. The effect of NO● donor SNAP on RyR1 activity in lipid bilayers was complex and dependent on several factors including, concentration, membrane potential and presence of agonists. Low concentrations of NO● donor SNAP increased RyR1 activity, whereas high concentrations (∼1 mM) caused channel inhibition (Hart and Dulhunty, 2000). In another study, a pretreatment of RyR1 with low concentrations of NO● donors prevented disulfide cross-linking induced by diamide. At the same time, NO● donors had no significant effect on the channel open probability. Higher doses of NO● donors, however, were able to increase RyR1 activity and induced disulfide cross-liking (Aghdasi et al., 1997). This type of complex regulation suggests the presence of several groups of functional thiol residues that differently modulate the RyR activity by RNS. Moreover, it has been shown that direct activation of RyR1 by submicromolar concentrations of NO● donors can only occur at physiological pO_2_ ∼10 mmHg (in contrast to ambient pO_2_ 150 mmHg). This suggests that RyR1 thiol residues have to be in the reduced state to be nitrosylated by NO● (Eu et al., 2000). However, these results could not be reproduced by another group. It was reported that NO● donors NOC-12 and GSNO activate RyR1 independently from pO_2_ (Cheong et al., 2005). Furthermore, the effect of NO● donors on RyR1 involved the CaM dissociation and S-nitrosylation at cysteine 3635 (Sun et al., 2003). In contrast to RyR1, RyR2 can be activated only by GSNO, but not NOC-12. S-nitrosylation of cysteine 3602 (corresponding to cysteine 3635 in RyR1) was not required for RyR2 activation by GSNO (Sun et al., 2008).

In cardiomyocytes, NOS1 and NOS3 can induce S-nitrosylation of different substrates, depending on their subcellular localization (Lima et al., 2010). NOS3 is localized within the caveolae together with β-adrenergic receptors and LTCCs. NOS1 resides to the SR, where it modulates the activity of RyR2 (Barouch et al., 2002). Study of RyR2 S-nitrosylation in NOS1^-/-^ mice has shown decreased S-nitrosylation, but increased oxidation of RyR2. Myocytes isolated from NOS1^-/-^ mice were characterized by increased SR Ca leak, decreased SR Ca load and higher propensity of spontaneous Ca waves (Gonzalez et al., 2007). It has been also shown that NOS1 inhibition leads to the development of ventricular arrhythmias under condition of elevated cytosolic [Ca]. The molecular mechanism of arrhythmias has been attributed to the combination of decreased RyR2 S-nitrosylation and increased RyR2 oxidation. Under condition of NOS1 inhibition, the XO inhibitor allopurinol or the NO● donor GSNO could prevent ventricular arrhythmias. Interestingly, NOS1 inhibition was associated with decreased RyR2 phosphorylation at the CaMKII site. Moreover, the combination of NOS1 inhibition and oxidative stress had an additive effect, producing severe arrhythmical phenotype (Cutler et al., 2012). In another study of NOS1^-/-^ myocytes, however, the decreased level of RyR2 S-nitrosylation was associated with decreased SR Ca release. NOS1 knockout or NOS inhibition reduced Ca spark frequency and SR Ca leak. Also, single channel recording revealed decreased open probability of RyR2 in the NOS1^-/-^ mice. The NO● donor SNAP was capable to reverse the observed effects of NOS1 inhibition (Wang et al., 2010). The discrepancy between these two studies may be explained by the complex interplay between S-nitrosylation and oxidation in the regulation of RyR2 activity. It seems that physiological levels of RyR2 S-nitrosylation can be necessary for maintaining the proper channel activity in the healthy heart. Under conditions of oxidative stress, however, S-nitrosylation may function as a protective mechanism against irreversible thiol oxidation and RyR2 dysfunction. Thus, de-nitrosylation of RyR2 would inhibit the channel activity in control conditions and activate it during oxidative stress.

## RYR Intersubunit Disulfide Cross-Linking

The idea of intersubunit cross-linking has been first introduced for RyR1 (Abramson and Salama, 1989) and then further confirmed for RyR2 (Mazurek et al., 2014). It has been suggested that intersubunit disulfide bond formation leads to structural rearrangements of the channel that cause SR Ca leak (Figure 2). Structural aspects of disulfide cross-linking in RyR1 have been extensively characterized in several studies (Moore et al., 1999; Zhang et al., 2003; Aracena-Parks et al., 2006). A cryo-EM analysis of RyR1 oxidation by H_2_O_2_ revealed a disulfide cross-linking together with significant changes in the channel morphology (Han et al., 2006). These data also revealed that disulfide bonds can be formed between subunits within the RyR1 complex rather than between different RyR1 tetramers. Multiple studies of RyR1 have suggested that cysteine 3635 can be involved in the disulfide cross-linking and the CaM binding. However, recent Cryo-EM analysis of RyR1 and RyR2 did not provide a structural basis for the possible disulfide formation in this region (Yan et al., 2015; des Georges et al., 2016; Wei et al., 2016; Dhindwal et al., 2017). It has been suggested that disulfide bond formation may take place between N-terminal regions of RyR2 subunits (Zissimopoulos et al., 2013). Alternatively, RyR cross-linking may occur not only between subunits within one channel, but also between subunits of different RyR channels. It is well-known that skeletal and cardiac muscle RyRs are arranged as large clusters (Franzini-Armstrong et al., 1999; Cabra et al., 2016). Given the tight packing of RyRs in the cluster, there is a possibility of disulfide cross-linking between subunits of two neighboring channels (Figure 2). However, there are no direct data demonstrating this mechanism.

Because the intersubunit interactions within the RyR2 complex dictate the channel gating (Orlova et al., 1996; Serysheva et al., 2008; Tung et al., 2010), any post-translational modifications that affect an interaction between RyR2 subunits should have a significant impact on the RyR2 function. We found a positive correlation between RyR2 cross-linking and SR Ca leak, suggesting that the post-translational modification is a strong regulator of RyR2. We have also reported that RyR2 oxidation and the intersubunit cross-linking play an important role in activation of SR Ca leak during oxidative stress (Mazurek et al., 2014) and generation of pro-arrhythmogenic Ca waves during excessive adrenergic activation (Bovo et al., 2015). Furthermore, a significant level of RyR2 cross-linking was observed in ventricular myocytes isolated from the rabbit HF model (Bovo et al., 2018a). This RyR2 modification contributed to an increase of SR Ca leak and the blunted force**-**frequency response of the failing heart.

## Calmodulin Dissociation During Oxidative Stress

CaM acts as an important regulatory protein of RyR2 that inhibits channel activity under both low and high [Ca] (Balshaw et al., 2001). During oxidative stress, increased levels of GSSG can decrease the binding affinity of apo-CaM and Ca-CaM to RyR2 (Figure 2). This would relieve the inhibitory effect of CaM on the RyR2 activity (Balshaw et al., 2001). Experiments in permeabilized ventricular myocytes have shown that RyR2 oxidation by H_2_O_2_ caused an alteration of the channel structure toward its arrhythmogenic unzipped conformation. This abnormal RyR2 conformation decreased the binding affinity to CaM, which can be restored by the RyR antagonist dantrolene (Oda et al., 2015). At the same time, oxidation caused no effect on the FKBP12.6 binding to RyR2. Dissociation of CaM from RyR2 due to the defective inter-domain interaction (or unzipping) has been linked to the increased SR Ca leak in HF (Ono et al., 2010). It has been shown that CaM isoforms with increased binding affinity to RyR2 have a potential to rescue the aberrant SR Ca release in HF (Hino et al., 2012). Furthermore, a recombinant CaM with decreased rate of dissociation from its binding domain on RyR2 unveiled anti-arrhythmogenic properties. In vivo gene delivery of this recombinant CaM into the heart partially restored RyR2 refractoriness and decrease a chance of arrhythmias in the catecholaminergic polymorphic ventricular tachycardia (CPVT) model caused by calsequestrin mutation (Liu et al., 2018).

## RYR2 Phosphorylation and Oxidative Stress

At least three functionally important phosphorylation sites have been identified in RyR2, including two PKA specific sites (serine 2030 and 2808) and one CaMKII site (serine 2814) (Marx et al., 2000; Xiao et al., 2006; Marx and Marks, 2013). RyR2 phosphorylation by PKA occurs in response to β**-**adrenergic receptor activation. Epinephrine and norepinephrine binding to β-adrenergic receptors increases adenylyl cyclase activity and cAMP production, following by PKA activation (El-Armouche and Eschenhagen, 2009). It has been shown that RyR2 phosphorylation at serine 2808 increased RyR2 open probability and caused FKBP12.6 dissociation from the channel (Marx et al., 2000; Figure 2). However, studies from different laboratories have yielded conflicting results regarding the role of RyR2 phosphorylation by PKA in HF (Benkusky et al., 2007; Shan et al., 2010a,b; Zhang et al., 2012). Interestingly, increased levels of RyR2 oxidation and S-nitrosylation have been reported in transgenic mice with the RyR2 mutation mimicking the constitutively phosphorylated serine 2808 (S2808D). While RyR2 activity in the S2808D mutant was normal at young age, in older animals the RyR2 displayed increased SR Ca leak caused by its oxidation and S-nitrosylation together with depletion of FKBP12.6 and other regulatory proteins (Shan et al., 2010a). These results were in line with another study in which mutation of one of two RyR2 phosphorylation sites (serine 2808 or 2814) reduced both oxidative stress and SR Ca leak in the mouse model of Duchenne muscular dystrophy (DMD) (Wang et al., 2015). Earlier studies have shown that DMD patients can develop ventricular arrhythmias associated with leaky RyR2 due to S-nitrosylated and FKBP12.6 depletion (Fauconnier et al., 2010).

While the functional significance of RyR2 phosphorylation has been debated, it is important to highlight that both CaMKII and PKA are sensitive to the intracellular redox state (Johnston et al., 2015; Figure 2). In contrast to the cAMP-dependent PKA activation, PKA activation by oxidative stress is associated with the disulfide bond formation between two regulatory PKA RIα subunits. In the proposed model, the disulfide cross-linking of two RIα subunits leads to the increased affinity of PKA to AKAP, that promotes subcellular targeting of the kinase catalytic subunit to the corresponding substrate (Brennan et al., 2006). Similarly to PKA, CaMKII can be also activated by oxidative stress (Erickson et al., 2008). The mechanism of ROS-dependent CaMKII activation required an initial activation by Ca-CaM followed by oxidation of methionine 281 and 282 yielding a persistent activation even after Ca-CaM dissociation. The mechanism of CaMKII activation by ROS was independent from the previously described activation by autophosphorylation. In *in vivo* experiments, NOX activation by angiotensin II (AngII) led to CaMKII oxidation. This activation was not observed in the p47^-/-^ transgenic mice lacking NOX activity (Erickson et al., 2008).

Since HF is commonly associated with oxidative stress (Mak and Newton, 2001), CaMKII activity is expected to be upregulated during this pathology. Once activated by ROS, CaMKII can contribute to arrhythmogenesis in HF by activating late sodium current (Wagner et al., 2011). In another study, a short-termed exposure of myocytes to H_2_O_2_ has resulted in the prolonged activation of CaMKII and long-termed facilitation of LTCC through mechanisms of CaMKII autophosphorylation and oxidation (Song Y.H. et al., 2011). Consistent with these findings, it has been shown that the pharmacological inhibition of CaMKII prevents arrhythmias induced by oxidative stress (Xie et al., 2009). The upregulation of CaMKII has been associated with the development of atrial fibrillation (AF). It has been shown that RyR2 phosphorylation at the CaMKII site was increased in AF patients, whereas RyR2 expression level was decreased. Cardiomyocytes from AF patients were characterized by increased SR Ca leak, which can be normalized by the CaMKII inhibitor KN93 (Neef et al., 2010). Moreover, it has been shown that NOX activation with AngII caused increased ROS production and CaMKII oxidation followed by an increase in Ca spark frequency. At the same time, the transgenic mice lacking the CaMKII phosphorylation site on RyR2 (S2814A) were protected from AF induced by AngII (Purohit et al., 2013). It has been shown that I/R-mediated arrhythmias were associated with CaMKII-dependent phosphorylation of RyR2 (Said et al., 2011). I/R also increased levels of RyR2 reversible redox modifications: S-glutathionylation and S-nitrosylation (Becerra et al., 2016). Arrhythmogenic effect of cardiac glucosides has been also attributed to the increased ROS production and RyR2 oxidation (Ho et al., 2011). It has been shown later that cardiac glucosides activate NOX2-mediated ROS production that causes CaMKII activation, RyR2 phosphorylation and Ca waves (Ho et al., 2014).

## FKBP Dissociation During Oxidative Stress

While there is no evidence that RyR2 oxidation directly causes FKBP12.6 dissociation from the channel, oxidative stress still may affect the RyR2-FKBP12.6 interaction through the mechanism of RyR2 phosphorylation (Figure 2). RyR2 hyper-phosphorylation at the PKA site has been shown to promote FKBP12.6 dissociation from RyR2 resulting in the increased channel activity during HF. It was suggested that the RyR2 hyper-phosphorylation was a result of PP1 dissociation from the RyR2 macromolecular complex (Marx et al., 2000). The interplay between RyR2 oxidation and FKBP12.6 dissociation has been studied in WT and the transgenic mice lacking the PKA phosphorylation site in RyR2 (the S2808A mice). In the WT mice excessive β-adrenergic stimulation caused increased FKBP12.6 dissociation from RyR2 in WT mice, but not in the S2808A mice. At the same time, the levels of RyR2 oxidation were increased in both WT and S2808A mice. To study the effects of oxidative stress and phosphorylation on the RyR2-FKBP12.6 interaction, SR vesicles were treated either with H_2_O_2_ or with H_2_O_2_ combined with CaMKII or PKA. Both H_2_O_2_ alone or in combination with CaMKII showed no significant effect on the FKBP12.6 dissociation from RyR2. However, RyR2 oxidation together with PKA caused almost complete FKBP12.6 dissociation. Chronic treatment of WT mice with isoproterenol increased levels of PKA-RyR2 phosphorylation, FKBP12.6 depletion and RyR2 oxidation, whereas in the S2808A mice these effects of adrenergic stimulation were partially abolished (Shan et al., 2010b).

## Conclusion

The vast body of evidence demonstrates a direct link between oxidative stress, RyR2 oxidation and increased SR Ca leak in several cardiac pathologies, including HF and MI. Molecular mechanisms of the impaired SR Ca handling can include structural and functional changes in the RyR2 complex due to thiol redox-modifications. However, effects of oxidative stress on RyR2 may go beyond the direct redox-modification of the channel and can involve dissociation of regulatory proteins and increased phosphorylation by PKA and CaMKII. It appears that phosphorylation and redox modifications may have an additive effect on RyR2 function. The experiments in the non-ischemic canine HF model have shown that RyR2 phosphorylation and thiol oxidation contribute to the different stages of HF: RyR2 phosphorylation by CaMKII manifests on the early stages of HF with following RyR2 oxidation during later stages (Belevych et al., 2011). In another HF study, the increased SR Ca leak was associated with RyR2 hyper-phosphorylation on both PKA and CaMKII sites together with thiol oxidation (Walweel et al., 2017), suggesting a combined effect of multiple factors on RyR2 dysfunction in HF. Despite abundant amount of experimental data pointing on the importance of oxidative stress, many of clinical trials that used antioxidant for treatment of HF did not yield promising results (Mak and Newton, 2001; Giordano, 2005; Thomson et al., 2007). However, trials with the beta-blocker with antioxidant properties carvedilol have shown some superiority over conventional beta-blockers, which can be attributed to its antioxidant effect (Packer et al., 2001). At the same time, it has been shown that carvedilol may also modulate the function of RyR2 independently from its beta-blocking function or antioxidant activity, but through the direct action on RyR2 activity (Zhou et al., 2011). Nevertheless, strategies focused on restoring the RyR2 structural integrity during oxidative stress may have a high therapeutic potential. Thus, further insights into molecular basis of RyR2 redox regulation are essential for the development of specific and effective therapeutic strategies.

## Author Contributions

All authors (RN, EB, and AZ) contributed to studying of literature and writing of the manuscript. All authors have approved the version to be published.

## Conflict of Interest Statement

The authors declare that the research was conducted in the absence of any commercial or financial relationships that could be construed as a potential conflict of interest.
